# Optoelectronic Oscillator-Based Microwave Photonic 20× Frequency Multiplier with Low Phase Noise

**DOI:** 10.3390/mi16030317

**Published:** 2025-03-10

**Authors:** Shi Jia, Qifan Zhang, Tianhao Zhang, Jinlong Yu

**Affiliations:** School of Electronic Information Engineering, Tianjin University, Tianjin 300072, China; zqf2024234133@tju.edu.cn (Q.Z.); zhangtianhao@tju.edu.cn (T.Z.); yujinlong@tju.edu.cn (J.Y.)

**Keywords:** optical frequency multiplication, injection locking, microwave photonics, optoelectronic oscillator

## Abstract

This letter presents a scheme for obtaining a microwave photonic frequency multiplier with low phase noise, in which an optoelectronic oscillator (OEO) is integrated with a directly modulated laser (DML)-based injection-locking technique. The system achieves frequency multiplication factors of 10 and 20, producing 10.01009 and 19.99095 GHz microwave signals with high side-mode suppression ratios of 62.0 and 50.2 dB. The measured single-sideband phase noise values are −121.87 and −111.95 dBc/Hz@10 kHz, which are 34.9 and 31.0 dB lower than those of traditional electronic frequency multiplication methods for 1 GHz signals. By utilizing the nonlinear characteristics of the DML, combined with injection locking and the OEO system, this cost-effective scheme reduces the system complexity while enhancing the stability and phase noise performance, offering a highly efficient solution for microwave frequency multiplication.

## 1. Introduction

Microwave signal sources play pivotal roles in a wide range of applications, including telecommunications, radar systems, aerospace, remote sensing, and beyond [1,2]. These signals are essential for the functionality of modern communication networks [3]. The ability to generate stable microwave signals with low phase noise and high spectral purity is a fundamental requirement for applications demanding high performance and reliability [4]. However, achieving such signals with high stability and noise performance poses great challenges.

In many cases, directly generating microwave signals is prohibitively expensive and technologically demanding [5]. This is especially true for higher-frequency microwave signals, as the cost of high-performance electronic components greatly increases. To address this, frequency multiplication techniques are commonly employed, in which lower-frequency signals are electronically multiplied to achieve the desired output [6]. While frequency multiplication methods are widely used and offer a convenient solution, they have several drawbacks. The most significant of these is phase noise degradation. In traditional electronic systems, the phase noise of the output signal increases according to the $20\log_{10}N$, where N is the multiplication factor [7]. Additionally, electronic frequency multipliers are typically constrained by the bandwidth limitations of electronic components, resulting in narrow frequency bands and other performance restrictions, especially in high-frequency applications [8].

Photonic techniques offer a promising alternative to electronic frequency multiplication, as they provide several advantages over traditional electronic methods [9]. Photonics-based approaches facilitate the generation of microwave signals with substantially lower phase noise, broader frequency spectra, and enhanced system flexibility [10]. Unlike electronic methods, which are constrained by narrower frequency spectra and significant phase noise degradation at higher multiplication factors, photonic techniques exploit the narrow linewidth and high stability of optical sources. Photonic frequency multiplication, in particular, has demonstrated the ability to generate microwave signals with superior phase noise performance compared with electronic multipliers.

A variety of photonic frequency multiplication methods have been successfully implemented [11,12]. External modulation, in which an optical modulator imprints a microwave signal onto an optical carrier followed by optical frequency multiplication, is one prominent approach [13]. Nonlinear optical effects, such as stimulated Brillouin scattering (SBS) or four-wave mixing (FWM), have also been employed to achieve frequency multiplication [8]. These methods offer broad bandwidths, low phase noise, and immunity to electromagnetic interference, making them particularly attractive for applications in high-performance systems. However, these approaches often involve complex setups, precise alignment, and costly components, which limit their scalability and practicality in many applications.

In comparison, directly modulated lasers (DMLs) offer a simpler alternative for frequency multiplication while retaining the key advantages of photonic systems. DMLs provide lower system complexity and reduced costs, making them an attractive option for practical applications. However, due to their inherent nonlinearities, their use in high-frequency multiplication is generally limited by non-ideal phase noise characteristics.

In addition to photonic frequency multiplication, injection locking and optoelectronic oscillators (OEOs) offer distinct advantages in microwave signal generation. Injection locking refers to the process in which a laser or an oscillator is forced to lock onto the frequency of an external signal, leading to improved frequency stability and reduced phase noise. This technique has been used in combination with optical frequency multiplication to achieve highly stable microwave sources [14]. The use of OEOs, which combine the benefits of optical oscillators and microwave electronics, is another promising approach for high-performance microwave signal generation. OEOs offer several advantages, including low phase noise, high spectral purity, and the ability to generate microwave signals over a wide range of frequencies [15]. The integration of injection locking with OEOs has proven to be an effective way to generate wide-bandwidth, low-phase-noise microwave signals, which makes it an ideal choice for precision-sensitive applications such as radar, communications, and scientific measurements.

Several existing systems based on optoelectronic oscillators (OEOs) and photonic frequency multiplication techniques have shown excellent phase noise and side-mode suppression ratio (SMSR) performance. Researchers demonstrated a frequency-sextupled (24.18 GHz) dual-loop OEO and achieved phase noise of −115.3 dBc dBc/Hz@100KHz and SMSR of 18.5 dB through joint FBG-MZI filtering. Despite eliminating electrical bandpass filters (EBPFs), the system required six polarization-critical components, demanding precise alignment and thermal stabilization [16]. Another study utilizes a single DP-QPSK modulator for both oscillation and quadrupling. This design achieved phase noise of −100 dBc/Hz@10KHz and SMSR of 19.6 dB. The need for exact modulator biasing (e-PM mode) and polarization-maintaining fiber loops introduced setup complexity [17]. By cascading two modulators for 4×, 6× and 8× multiplication, another approach demonstrated phase noise of −113 dBc/Hz@100KHz with an SMSR value of 20 dB but required synchronized bias control of both modulators [11].

This letter presents an OEO-based approach to achieve frequency multiplication with low-phase noise via an injection locking technique based on a directly modulated laser (DML). The proposed system achieves frequency multiplication factors of 10 and 20 and produces stable microwave signals with low phase noise and high side-mode suppression ratios (SMSRs). The experimental setup utilizes a directly modulated laser for injection locking, combined with an optoelectronic oscillator to exploit the nonlinear characteristics of DMLs. The proposed system reduces complexity and cost while delivering superior performance by simplifying the frequency multiplication process compared with external modulation methods. The experimental results show that our system achieves impressive improvements in both phase noise and SMSR, with measured single-sideband (SSB) phase noise values of −121.87 and −111.95 dBc/Hz@10 kHz for 10 GHz and 20 GHz signals, respectively. This represents a phase noise reduction of 34.9 dB and 31.0 dB compared to traditional electronic frequency multipliers. Furthermore, our system achieves high SMSRs of 62.0 dB and 50.2 dB at the same frequencies, which are comparable to the best photonic OEO systems, while maintaining a notably streamlined architecture. Compared with the aforementioned methods, our system achieves a higher frequency multiplication factor of 20× while exhibiting improved phase noise and enhanced SMSR performance through a simplified architecture. Besides, the proposed method holds great potential for applications in advanced communication systems, radar systems, and photonic signal processing, offering a cost-effective and high-performance solution for generating 10× and 20× frequency-multiplied microwave signals with low phase noise and high spectral purity.

## 2. Materials and Methods

DMLs represent one of the most straightforward and cost-effective tools for generating modulated optical signals. In a DML, the modulation is directly applied to the driving current of the laser, causing both amplitude and frequency modulation of the emitted optical signal. Compared with external modulation schemes, this direct modulation process makes DMLs particularly attractive in terms of simplicity and reduced system complexity. External modulators, such as Mach–Zehnder modulators (MZMs), typically require additional components for biasing, optimization, and linearization, which increases both the cost and complexity of the system. Furthermore, while external modulation can offer a high degree of linearity, DMLs provide a more economical alternative with a simpler design.

However, DMLs also exhibit inherent limitations, particularly in the generation of higher-order harmonic components. A key limitation is the chirp effect, which is more pronounced in DMLs than in external modulators. This chirp, which arises from the modulation of the driving current, induces frequency variations proportional to the amplitude modulation, thereby contributing to the generation of higher-order harmonics in the output spectrum. Unlike external modulators, which typically suppress harmonics beyond the third order, DMLs produce a much broader range of harmonics. This broader harmonic spectrum can lead to pronounced sideband emissions, resulting in a higher degree of nonlinearity and the introduction of larger-amplitude fluctuations into the sidebands. While such nonlinearity is often considered a drawback, it can be advantageously exploited in specific applications, particularly those in which higher-order harmonics are utilized for frequency multiplication.

To illustrate the DML principle, consider a simple sinusoidal input signal with angular frequency $\omega_{m}$. The modulation applied to the driving current of the DML induces both amplitude and frequency modulation of the optical signal. The resulting output electric field can be expressed as follows [18]:

(1) $$E\left(t\right)=E_{0}\left[1+\mathrm{mcos}\left(\omega_{m}t\right)\right]\exp\left[j\left(\omega_{0}t+\beta \cos\left(\omega_{m}t\right)\right)\right]$$

where $E_{0}$ is the amplitude of the unmodulated optical signal emitted by the DML, m is the modulation index, which determines the extent of the amplitude modulation, and β represents the frequency modulation index, which determines the extent of the frequency modulation. The frequency $\omega_{m}$ corresponds to the modulation frequency, and $\omega_{0}$ is the central angular frequency of the unmodulated laser.

By applying the Jacobi–Anger expansion to (1), we have:

(2) $$\begin{array}{cc} E\left(t\right) & =E_{0}\left[1+\mathrm{mcos}\left(\omega_{m}t\right)\right]e^{\left[j\left(\omega_{0}t+\beta \cos\left(\omega_{m}t\right)\right)\right]}\\ & =E_{0}\left[1+\mathrm{mcos}\left(\omega_{m}t\right)\right]\left(cos\left(\omega_{0}t\right)+j\sin\left(\omega_{0}t\right)\right)\\ & \;\cdot \left[J_{0}\left(\beta \right)+2\sum_{n=1}^{\infty }{\left(j\right)}^{n}J_{n}\left(\beta \right)cos\left({n\omega }_{m}t\right)\right]\\ & =E_{0}\cdot J_{0}\left(\beta \right)\left[1+\mathrm{mcos}\left(\omega_{m}t\right)\right]\left(cos\left(\omega_{0}t\right)+j\sin\left(\omega_{0}t\right)\right)+{2E}_{0}\left[1+\mathrm{mcos}\left(\omega_{m}t\right)\right]\\ & \;\cdot \sum_{n=1}^{\infty }{\left(j\right)}^{n}J_{n}\left(\beta \right)cos\left({n\omega }_{m}t\right)\left(cos\left(\omega_{0}t\right)+j\sin\left(\omega_{0}t\right)\right)\\ & =E_{0}\cdot J_{0}\left(\beta \right)\left[1+\mathrm{mcos}\left(\omega_{m}t\right)\right]\left(cos\left(\omega_{0}t\right)+j\sin\left(\omega_{0}t\right)\right)+E_{0}\left[1+\mathrm{mcos}\left(\omega_{m}t\right)\right]\\ & \;\cdot \sum_{n=1}^{\infty }{\left(j\right)}^{n}J_{n}\left(\beta \right)\left[cos\left(\left({\omega_{0}+n\omega }_{m}\right)t\right)+cos\left(\left({\omega_{0}-n\omega }_{m}\right)t\right)\right]\\ & \;+E_{0}\left[1+\mathrm{mcos}\left(\omega_{m}t\right)\right]\\ & \;\cdot \sum_{n=1}^{\infty }{\left(j\right)}^{n+1}J_{n}\left(\beta \right)\left[sin\left(\left({\omega_{0}+n\omega }_{m}\right)t\right)+sin\left(\left({\omega_{0}-n\omega }_{m}\right)t\right)\right]\\ & =E_{0}\cdot J_{0}\left(\beta \right)\left[1+\mathrm{mcos}\left(\omega_{m}t\right)\right]e^{j\omega_{0}t}\\ & \;+E_{0}\left[1+\mathrm{mcos}\left(\omega_{m}t\right)\right]\cdot \\ & \;\sum_{n=1}^{\infty }{\left(j\right)}^{n}J_{n}\left(\beta \right)\left[e^{j\left({\omega_{0}+n\omega }_{m}\right)t}+e^{j{(\omega_{0}-n\omega }_{m})t}\right] \end{array}$$

The nth harmonic frequency term can thus be obtained. The selection of harmonic orders is based on a trade-off between system performance and the achievable signal frequency. Lower harmonic orders may improve signal quality, such as phase noise and side-mode suppression, but they may not be sufficient for generating higher-frequency signals without the use of very high-frequency RF sources. On the other hand, higher harmonic orders can generate higher-frequency signals but often degrade performance, including increased phase noise and reduced SMSR. The 10× and 20× harmonic orders in our system were selected as they provide a good balance between achieving high-frequency multiplication and maintaining good signal quality. These harmonic orders are also commonly used in photonic frequency multiplication systems, making them both practical and representative of similar systems. By leveraging these higher-order harmonics, injection locking can be used to stabilize and amplify the signal at integer multiples of the original frequency.

Injection locking occurs when the phase of a free-running oscillator is synchronized to that of an injected signal, which results in the locking of the oscillator frequency to the injected signal frequency. For a DML, the n-th harmonic frequency generated due to the direct modulation of the laser can be expressed as follows:

(3) $$\omega_{n}=n\cdot \omega_{m}$$

where n is the harmonic order, the frequency of the injected signal must be within a certain range, known as the injection locking range, to ensure stable phase synchronization between the DML output and the injected signal. The injection locking range $\Delta \omega_{lock}$ for a distributed feedback (DFB) laser can be described by the following expression [19]:

(4) $$\Delta \omega_{lock}=\frac{\omega_{0}}{Q}\frac{P_{in}}{P_{th}}$$

where $\omega_{0}$ is the central angular frequency of the unmodulated DML, Q is the quality factor of the DFB laser, $P_{in}$ is the injected power, and $P_{th}$ is the threshold power required to initiate locking. The injection locking range determines the allowable frequency detuning between the injected signal and the DFB output for successful phase locking. The nth frequency of the DML must fall within the injection locking range for synchronization to occur. In this case, the nth harmonic frequency lies within the locking range if:

(5) $$\left|\omega_{n}-\omega_{0}\right|\leq \Delta \omega_{lock}$$

Once injection locking is achieved, the output of the DFB laser forms a coherent optical frequency comb, with the nth harmonic frequencies corresponding to the desired multiplication factor. This optical frequency comb, which consists of multiple equally spaced frequency components, can now be input into an OEO system. The OEO system can convert the optical frequency comb into a corresponding electrical frequency comb. Upon passing this comb through a bandpass filter, the desired frequency components corresponding to the specific multiplication factor, such as 10× or 20×, can be isolated. These filtered components correspond to the nth harmonics of the original optical signal, as described in Equation (1). The phase noise of the microwave signal generated by the OEO can be expressed as follows [20]:

(6) $$\begin{array}{c} \langle {\left|S_{locking}\left(\omega \right)\right|}^{2}\rangle =\frac{\cos^{2}{(n\varphi }_{0})}{\cos^{2}{(n\varphi }_{0})+{\left(\frac{\omega }{\Delta \omega_{lock}}\right)}^{2}}\langle {\left|S_{input}\left(\omega \right)\right|}^{2}\rangle \\ +\frac{{\left(\frac{\omega }{\Delta \omega_{lock}}\right)}^{2}}{\cos^{2}{(n\varphi }_{0})+{\left(\frac{\omega }{\Delta \omega_{lock}}\right)}^{2}}\langle {\left|S_{free}\left(\omega \right)\right|}^{2}\rangle \end{array}$$

where $\langle \left|S_{locking}\left(\omega \right)\right|\rangle$ is the SSB phase noise of the output signal for the injection-locked OEO, $\langle \left|S_{input}\left(\omega \right)\right|\rangle$ is the SSB phase noise of the input signal, and $\langle \left|S_{free}\left(\omega \right)\right|\rangle$ is the SSB phase noise of the output signal for the free-running OEO. $\varphi_{0}$ is the constant phase difference between the injected and locked signals.

The output electrical signal, with stable and low phase noise characteristics, represents the frequency-multiplied microwave signal. This process allows the efficient generation of microwave signals at high frequencies while maintaining the integrity of the signal phase and minimizing phase noise.

## 3. Results

### 3.1. Experiment Setup of Optical Frequency Multiplier

**Figure 1:**
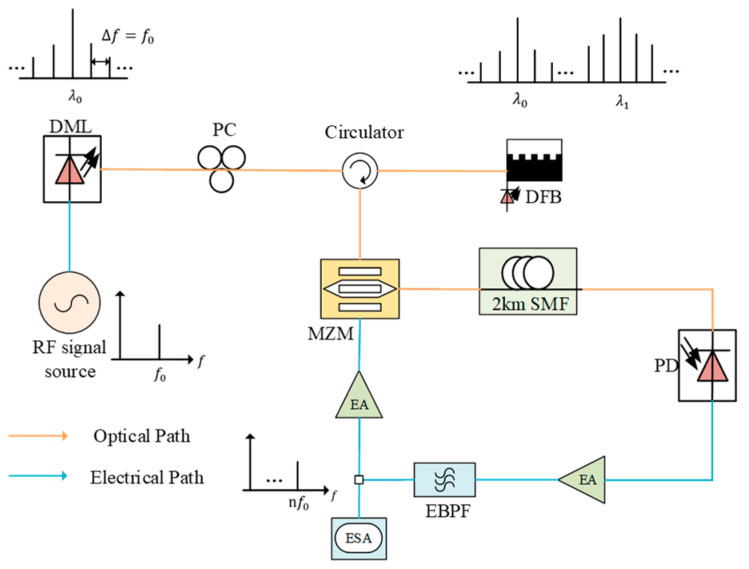
Block diagram of the proposed method. DFB: distributed feedback laser; PC: polarization controller; MZM: Mach-Zehnder modulator; SMF: single-mode fiber; PD: photodiode; EA: electrical amplifier; EBPF: electrical bandpass filter; ESA: electrical spectrum analyzer.

The experimental setup, as illustrated in Figure 1, was designed to validate the principle of the proposed microwave photonic frequency multiplication system by leveraging the unique properties of DMLs and injection locking in DFB lasers combined with an OEO.

The process begins with a 1 GHz electrical signal generated by an RF signal source (RIGOL DG1062 from China), which is applied as a driving signal to a DML with a wavelength of 1550.12 nm. The DML modulates the input microwave signal onto an optical carrier, producing an optical signal with strong harmonic components. This modulated optical signal is directed into a circulator, which guides it into a DFB laser for injection locking. The DFB laser achieves injection locking when a specific harmonic component of the optical signal generated by the DML falls within its injection locking range. Injection locking effectively suppresses unwanted frequency components and amplifies the desired harmonic. The injection-locked optical signal from the DFB laser, which is now stabilized in both phase and amplitude, is routed back through the circulator and divided into two paths. One serves as the input to an MZM, and the other is monitored by an electronic spectrum analyzer (Agilent 8564EC from America) and a phase noise analyzer (RS FSWP50 from German) during testing. The modulated optical signal then propagates through a 2 km single-mode optical fiber, which serves as an energy storage element, providing the delay critical for sustaining oscillation and ensuring phase stability within the loop. After passing through the fiber, the optical signal is converted into an electrical signal by a high-speed photodetector (PQW20A-L from China, with a bandwidth of 20 GHz). The resulting electrical signal is amplified by an electrical amplifier and then passed through a narrowband bandpass filter to select the desired frequency component, 10 GHz or 20 GHz, and eliminate excess frequencies caused by sideband noise. The EBPF used at 10 GHz has a bandwidth of 100 MHz, and the one at 20 GHz has a bandwidth of 150 MHz. The filtered electrical signal is split into two paths. One path is further amplified and routed back to the MZM, closing the OEO loop and maintaining stable oscillation. The other path is sent to an electrical spectrum analyzer and a phase noise analyzer for evaluation of the system performance. This feedback configuration in the OEO enables the generation of a high-purity microwave signal with excellent spectral selectivity and phase noise characteristics.

### 3.2. Frequency Multiplication Performance of the System

**Figure 2:**
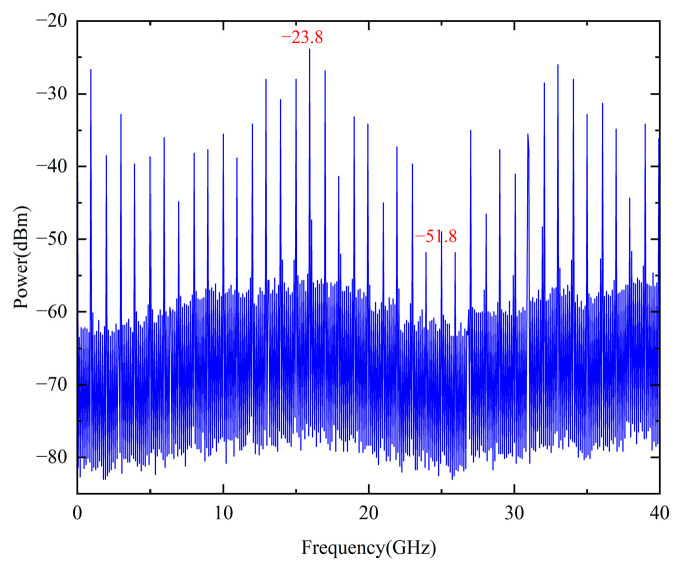
The electrical frequency spectrum of the generated electrical frequency comb.

The electrical frequency spectrum of the injection-locked DFB laser output was first analyzed to confirm successful injection locking. As shown in Figure 2, 40 frequency components are generated in the range from 1 GHz to 40 GHz. The comb spacing of the resulting spectrum is observed to be 1 GHz, indicating that the harmonic spacing is induced by the injection locking process. At this stage, the spectrum is not flat, and a noticeable difference in the peak power levels of the various components is observed. The highest spectral component has a peak value of −23.8 dB, whereas the lowest spectral component has a peak value of −51.8 dB. This power discrepancy could potentially influence phase noise performance and the side-mode suppression ratio (SMSR). While a flatter spectrum could be beneficial for certain applications, in this work, the primary objective is not to achieve a flattened spectrum but to ensure that the target frequency components, specifically at 10 GHz and 20 GHz, maintain sufficiently high power levels of effective extraction and processing in the subsequent OEO system. By employing appropriate filtering within the OEO system, the impact of power variation in the comb spectrum on the final multiplied signals can be minimized, ensuring stable and high-quality output at the desired frequencies.

**Figure 3:**
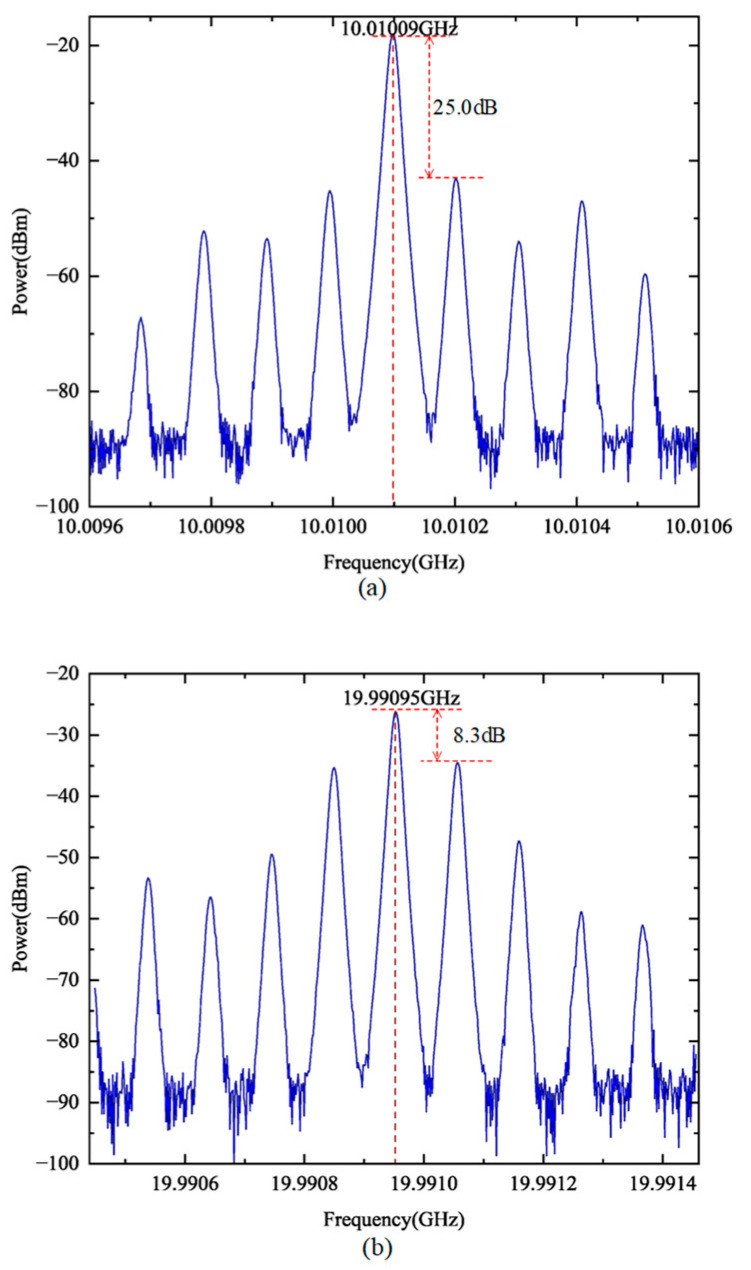
Electrical frequency spectrum of the free-running OEO at the center frequency of (**a**) 10 GHz and (**b**) 20 GHz.

The electrical frequency spectrum of the free-running OEO was then analyzed to evaluate the baseline oscillation characteristics of the system, in which the oscillation was initiated by noise and sustained through the feedback loop. In this mode, the OEO central frequency is determined by the bandpass filter. The results for the free-running OEO operation are shown in Figure 3, where the bandpass filter was configured for central frequencies of 10 GHz and 20 GHz, corresponding to the 10× and 20× frequency multiplication settings, respectively. The spectra in Figure 3 illustrate the spectra of the OEO output under free-running conditions. At 10× frequency multiplication, the central oscillation frequency is observed at 10.01009 GHz, with an associated SMSR of 25.0 dB, which indicates a poor suppression of side modes. Similarly, for the 20× frequency multiplication setting, the central oscillation frequency shifts to 19.99095 GHz, with an SMSR of 8.3 dB.

**Figure 4:**
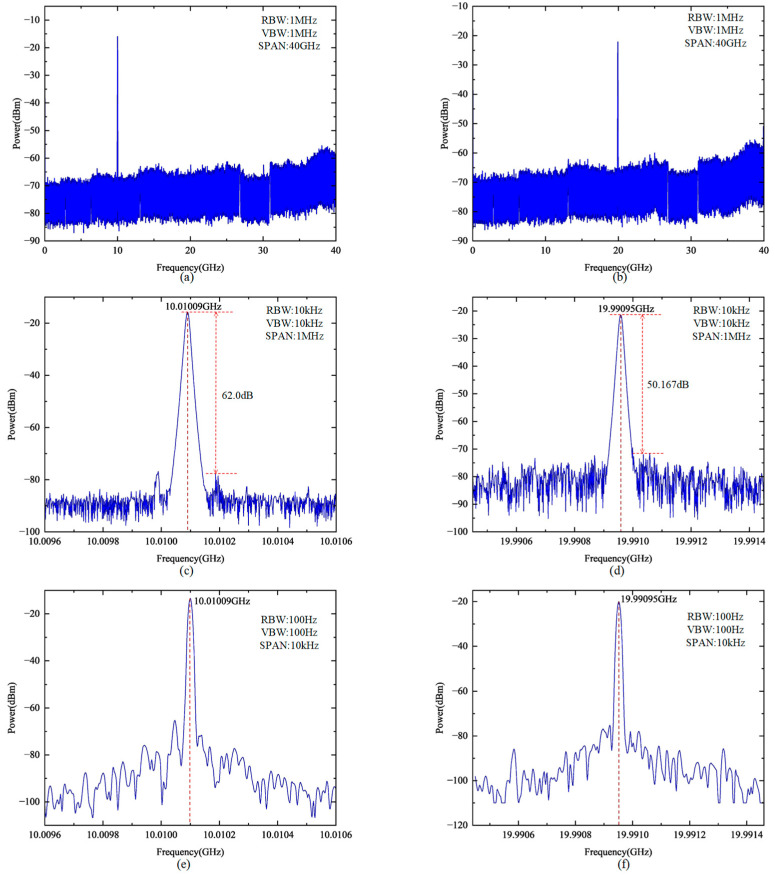
Electrical frequency spectra of the generated microwave signals: (**a**) 10× frequency multiplication signal with a resolution bandwidth (RBW) of 1 MHz, a video bandwidth (VBW) of 1 MHz, and a span of 40 GHz; (**b**) 20× frequency multiplication signal with an RBW of 1 MHz, a VBW of 1 MHz, and a span of 40 GHz; (**c**) 10× frequency multiplication signal with an RBW of 10 kHz, a VBW of 10 kHz, and a span of 1 MHz; (**d**) 20× frequency multiplication signal with an RBW of 10 kHz, a VBW of 10 kHz, and a span of 1 MHz; (**e**) 10× frequency multiplication signal with an RBW of 100 Hz, a VBW of 100 Hz, and a span of 10 kHz; (**f**) 20× frequency multiplication signal with an RBW of 100 Hz, a VBW of 100 Hz, and a span of 10 kHz.

The OEO was then operated under injection-locked conditions, with the injection-locked DFB laser signal serving as the input to the system. By adjusting the central frequency of the bandpass filter, the OEO was configured to generate the desired 10× and 20× frequency-multiplied signals. The corresponding spectra are shown in Figure 4. In Figure 4a,b, electrical spectra with a span of 40 GHz and a resolution bandwidth (RBW) of 1 MHz are presented, illustrating the overall OEO output. Figure 4c,d display spectra with a span of 1 MHz and an RBW of 10 kHz, while Figure 4e,f show spectra with a span of 10 kHz and an RBW of 100 Hz to provide a detailed view of the multiplied frequencies. As illustrated, under injection locking, the OEO output exhibits substantial suppression of side-mode components. For the 10× frequency multiplication setting, the central frequency peak is observed at 10.01009 GHz, with an SMSR of 62.0 dB. Similarly, under the 20× frequency multiplication setting, the central frequency peak shifts to 19.99095 GHz, and the SMSR is 50.2 dB. The high SMSR achieved for both frequency multiplication settings highlights the stability and spectral purity of the proposed system, making it well-suited for applications requiring precise and low-noise microwave signal generation.

**Figure 5:**
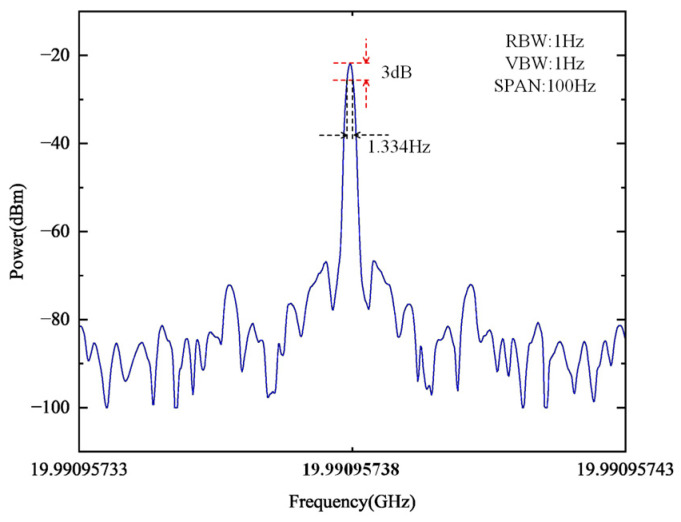
Electrical frequency spectra of the 20× frequency-multiplied signal with an RBW of 1 Hz, a VBW of 1 Hz, and a span of 100 Hz. The measured linewidth is approximately 1.334 Hz.

**Figure 6:**
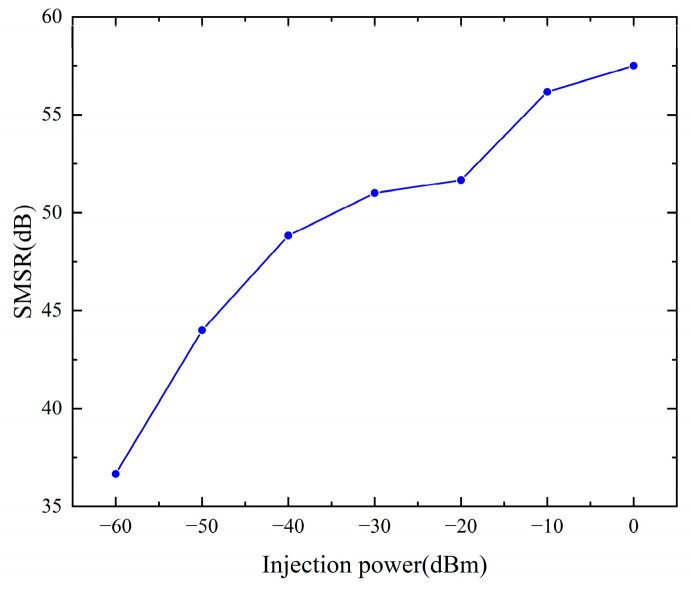
SMSR versus injection power for the 20× frequency-multiplied signal at 20 GHz.

In order to accurately characterize the linewidth of the multiplied signal, we measured the output spectrum under standard conditions with a span of 100 Hz and an RBW of 1 Hz. As shown in Figure 5, the measured linewidth is approximately 1.334 Hz. Furthermore, the impact of injection power on the SMSR was investigated to evaluate its influence on system performance. As the injection power increases, the locking range expands, leading to enhanced spectral purity and stronger suppression of side modes. This improvement in SMSR can be attributed to the more effective stabilization of the injected signal, which minimizes undesired frequency components. The results, as illustrated in Figure 6, indicate a monotonic increase in SMSR with higher injection power, demonstrating that stronger injection enhances side-mode suppression.

**Figure 7:**
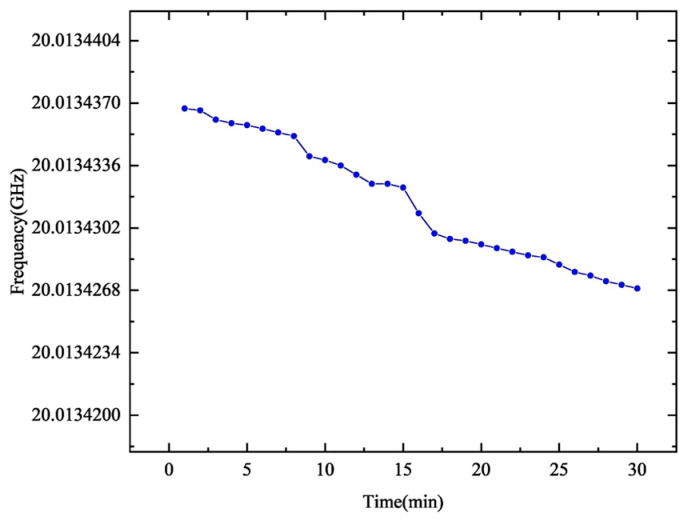
Long-term frequency stability evaluation of the 20× multiplied signal (20 GHz) over 30 min.

In addition, the frequency stability of the multiplied signal was evaluated by monitoring the drift over a 30-min period for the 20× frequency multiplication setting. As shown in Figure 7, the frequency drift was measured to be only 16 kHz over 30 min, which corresponds to a fractional stability of $8\times 10^{-7}$. These results confirm that the system exhibits excellent frequency stability, further validating its potential for high-performance applications.

**Figure 8:**
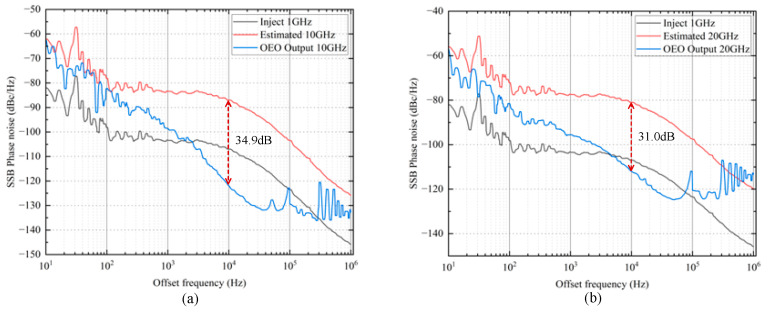
SSB phase noise: (**a**) Injected 1 GHz signal (black line); estimated 10 GHz signal (red line); OEO output 10 GHz signal (blue line). (**b**) Injected 1 GHz signal (black line); estimated 20 GHz signal (red line); OEO output 20 GHz signal (blue line).

Finally, a detailed phase noise comparison was conducted between the proposed optical frequency multiplication method and the traditional electronic frequency multiplication approach. In the experiments for 10× and 20× frequency multiplication, the phase noise of the original 1 GHz electrical signal was measured first, followed by the phase noise after frequency multiplication using the electrical method and the proposed optical method. In the electronic frequency multiplication method, the phase noise is inherently degraded by 20lgN dB, which corresponds to degradation levels of 20 dB and 26.02 dB for 10× and 20× frequency multiplication, respectively. The SSB phase noise for 10× and 20× frequency multiplication is shown in Figure 8. The proposed optical frequency multiplication method achieves the lowest phase noise, with values of −121.87 dBc/Hz@10 kHz and −111.95 dBc/Hz@10 kHz for 10× and 20× frequency multiplication, respectively. In contrast, the electronic frequency multiplication method results in phase noise values of −86.98 dBc/Hz@10 kHz and −80.96 dBc/Hz@10 kHz. The results clearly demonstrate the superior performance of the optical method. Compared with the electronic frequency multiplication method, the proposed approach achieves phase noise improvements of 34.9 dB for 10× frequency multiplication and 31.0 dB for 20× frequency multiplication at a 10 kHz offset. This demonstrates that the proposed optical frequency multiplication method successfully avoids phase noise deterioration and achieves a low phase noise.

Our proposed method utilizes the high nonlinearity of DMLs to generate stable optical frequency combs through injection locking. Additionally, we utilize the low-phase noise characteristics of the OEO system to achieve frequency multiplication with superior phase noise performance, i.e., compared with electronic frequency multiplication, the proposed microwave photonic approach achieves lower phase noise. Additionally, compared with MZM-based methods, DMLs offer a simpler setup, lower costs, and stronger nonlinear effects, enabling higher multiplication factors. These advantages are validated by the successful generation of 10× and 20× frequency-multiplied signals with superior spectral purity and phase noise performance. However, the current setup was constrained by the center frequency of the electrical bandpass filter (EBPF), which limited the maximum achievable frequency to 20 GHz. While only 10× and 20× frequency multiplications were experimentally demonstrated, the proposed method is theoretically scalable to even higher frequency signals. By selecting a higher base frequency and employing appropriately matched electrical amplifiers and bandpass filters within the OEO system, the desired harmonic components can be isolated for higher-frequency applications.

Furthermore, our system is not restricted to fixed multiplication factors such as 10× or 20×; by adjusting the RF source and modifying the corresponding filter center frequencies, a wide range of frequency-multiplied signals can be generated. For example, to generate a 14 GHz signal, we could use a 1 GHz base signal and choose a 14× multiplication factor, selecting 14 GHz filters and amplifiers for the system. Alternatively, a 2 GHz base signal could be used for 7× multiplication. Similarly, for a 17.3 GHz signal, we could use a 1.73 GHz base signal and multiply by 10, a 5.76 GHz base signal for 3× multiplication, or even a 1.33 GHz signal for 13× multiplication. This inherent flexibility enables the system to be adapted for various high-frequency microwave applications requiring continuous wideband signal generation.

## 4. Discussion

We have experimentally demonstrated an OEO-based method for generating 10× and 20× frequency-multiplied microwave signals by using a DML with the injection locking technique. This approach utilizes the nonlinear characteristics of the DML to generate a rich harmonic comb, which is stabilized through injection locking with a DFB laser. The stabilized optical signal is then processed by the OEO system to produce high-purity, low-phase-noise microwave signals.

Our experimental results show that the frequency-multiplied signals at 10 GHz and 20 GHz exhibit SMSRs of 62.0 dB and 50.2 dB, respectively. Moreover, the SSB phase noise of the multiplied signals is measured as −121.87 dBc/Hz@10 kHz and −111.95 dBc/Hz@10 kHz for the 10 GHz and 20 GHz signals, respectively, which represent improvements of 34.9 dB and 31.0 dB over the phase noise performance of conventional electronic frequency multiplication methods. The proposed method holds great potential for achieving even higher frequency multiplication factors using filters with higher center frequencies, and the inherent flexibility of our approach enables the generation of continuous wideband signals, broadening its applicability across diverse high-frequency applications. Furthermore, due to the lower complexity of our system compared to conventional methods, it offers a practical solution for integration into microwave communication systems as a signal source. This makes it an attractive alternative for applications requiring low-phase-noise, high-purity signal generation, providing a compact, cost-effective, and reliable choice for next-generation communication technologies.

## Data Availability

The data presented in this study are available from the corresponding author upon reasonable request.
